# Advances in large viewing angle and achromatic 3D holography

**DOI:** 10.1038/s41377-024-01468-4

**Published:** 2024-06-07

**Authors:** Jiaming Huang, Yu Chen, Guixin Li

**Affiliations:** 1 Department of Materials Science and Engineering, Southern University of Science and Technology, Shenzhen, 518055 China; 2 Institute for Applied Optics and Precision Engineering, Southern University of Science and Technology, Shenzhen, 518055 China

**Keywords:** Displays, Liquid crystals

## Abstract

Optical holography is a promising technique to achieve a naked-eye 3D display. However, the narrow viewing angle and chromatic aberration are the two key issues that usually limit the holographic display performance. A recent work proposes a novel way to circumvent these constraints by introducing a color liquid crystal grating into a time-sequenced holography system.

Holography, firstly proposed by D. Gabor in 1948 to solve the image aberration problem of electron microscopes^1^, is a technique through which we are able to record and reconstruct the wavefront information of 3D objects. Along with the fast development of laser technology and computer-generated holography (CGH)^2^, the concept of colorful holographic display was proposed. However, all the diffractive optical elements, including liquid crystal-based spatial light modulators (SLMs), exhibit strong dispersion effects. This means that the holographic images at different illumination wavelengths have different sizes. Therefore, it has been challenging to realize achromatic holographic display.

In imaging applications, the dispersion effects from diffractive optics can be greatly reduced by designing achromatic metalens^3,4^ and multi-level diffractive lens^5^. However, a similar concept is difficult to be applied to the fields of holographic display. In 2003, the time division multiplexing method was proposed and used to demonstrate the bi-color holographic display^6^. After about two decades, people were able to demonstrate real-time 3D colorful holography by using large-scale hologram datasets and convolutional neural network method^7^. Later, the model-driven neural network method^8,9^ was developed to reduce the requirement of hologram datasets and realize full-color and high-fidelity CGH. By further introducing a liquid lens into the holographic display system, the real-time 3D scene hologram acquisition was also successfully demonstrated^9^.

It should be noted that the viewing angle in holographic displays relies on the pixel size of the holograms. In the metasurface diffractive optical elements, the pixel size is usually at the sub-wavelength scale, therefore the viewing angle of a corresponding holographic image is up to 60 degrees^10^. In comparison, the viewing angle of the conventional SLM-based holographic display is usually less than 10 degrees, which will definitely limit the practical application of related technologies. To overcome this obstacle, Qionghua Wang’s group from Beihang University proposes to introduce a color liquid crystal grating into the time-sequenced holographic system.

In the recent work published in *Light: Science & Applications*^11^, Di Wang et al. demonstrated a chromatic aberration-free 3D holographic display system with a viewing angle of about 50 degrees. As shown in Fig. 1, the display system mainly consists of three sections: the red, green, and blue (RGB) laser sources, an SLM, and a color liquid crystal grating. The working principle is briefly summarized as follows. Firstly, a target 3D image is decomposed into the RGB channels, and the computer-generated holograms of the three channels are then calculated. By controlling the time sequence of the laser output and the holograms on the SLM, the holographic images in the RGB channels can be displayed with negligible chromatic aberration effect. The diffraction angles of the RGB images can be further controlled by superposing the geometric phase gradients into the hologram patterns on the SLM. Secondly, a self-developed color liquid crystal grating is inserted into the optical path to enlarge the viewing angles of the holographic images through a cascaded diffraction process. The color liquid crystal grating is composed of the RGB sub-gratings which have pre-defined periods. When the RGB holographic images pass through the corresponding sub-gratings, they propagate to the different diffraction orders.

**Fig. 1:**
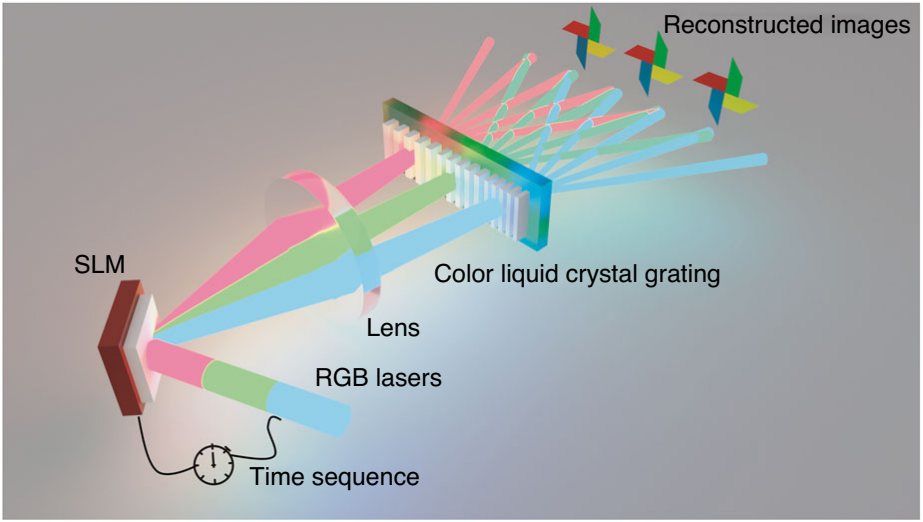
Concept of the achromatic holography with large viewing angle. The red, green, and blue lasers are incident on the time-sequenced hologram patterns on the SLM, and the RGB holographic images are projected on the three predefined sections on the color liquid crystal grating. The RGB images are further diffracted to multiple diffraction orders. Finally, the achromatic holographic display with a large view angle is demonstrated

As the periods of the RGB sub-gratings are much larger than the wavelength of visible light, one can see multiple diffraction orders. To overlap the RGB holographic images at the required diffraction direction, the incident angles should match well with the grating periods. By judiciously controlling the applied voltage on the color liquid crystal grating, the diffraction efficiency at each order is almost the same as each other. Finally, the authors are able to synthesize colorful 3D holographic images at seven diffraction orders with the largest viewing angle of about 50 degrees. Based on the human eye’s persistence effect, the colorful holographic images can be seen at the same time as long as the refreshing rate of SLM is fast enough.

In summary, the color liquid crystal grating-based holographic display system provides a novel and feasible solution to realize large viewing angles and achromatic 3D holography. The viewing angle of the holographic image can be further increased by reducing the pitch size of both SLM and the color liquid crystal grating. The demonstrated achromatic holography may have important applications in multi-channel bio-imaging, parallel optical information processing, and AR/VR applications.
